# Downregulation of MicroRNA-9 in iPSC-Derived Neurons of FTD/ALS Patients with TDP-43 Mutations

**DOI:** 10.1371/journal.pone.0076055

**Published:** 2013-10-15

**Authors:** Zhijun Zhang, Sandra Almeida, Yubing Lu, Agnes L. Nishimura, Lingtao Peng, Danqiong Sun, Bei Wu, Anna M. Karydas, Maria C. Tartaglia, Jamie C. Fong, Bruce L. Miller, Robert V. Farese, Melissa J. Moore, Christopher E. Shaw, Fen-Biao Gao

**Affiliations:** 1 Department of Neurology, University of Massachusetts Medical School, Worcester, Massachusetts, United States of America; 2 Department of Clinical Neuroscience, Institute of Psychiatry, London, United Kingdom; 3 Department of Biological Chemistry and Molecular Pharmacology, University of Massachusetts Medical School, Worcester, Massachusetts, United States of America; 4 Howard Hughes Medical Institute, Worcester, Massachusetts, United States of America; 5 Gladstone Institute of Cardiovascular Disease, San Francisco, California, United States of America; 6 Center for Neurologic Diseases, Harvard Medical School, Boston, Massachusetts, United States of America; 7 Memory and Aging Center, Department of Neurology, University of California San Francisco, San Francisco, California, United States of America; 8 Departments of Medicine and Biochemistry and Biophysics, University of California San Francisco, San Francisco, California, United States of America; Louisiana State University Health Sciences Center, United States of America

## Abstract

Transactive response DNA-binding protein 43 (TDP-43) is a major pathological protein in frontotemporal dementia (FTD) and amyotrophic lateral sclerosis (ALS). There are many disease-associated mutations in TDP-43, and several cellular and animal models with ectopic overexpression of mutant TDP-43 have been established. Here we sought to study altered molecular events in FTD and ALS by using induced pluripotent stem cell (iPSC) derived patient neurons. We generated multiple iPSC lines from an FTD/ALS patient with the *TARDBP* A90V mutation and from an unaffected family member who lacked the mutation. After extensive characterization, two to three iPSC lines from each subject were selected, differentiated into postmitotic neurons, and screened for relevant cell-autonomous phenotypes. Patient-derived neurons were more sensitive than control neurons to 100 nM straurosporine but not to other inducers of cellular stress. Three disease-relevant cellular phenotypes were revealed under staurosporine-induced stress. First, TDP-43 was localized in the cytoplasm of a higher percentage of patient neurons than control neurons. Second, the total TDP-43 level was lower in patient neurons with the A90V mutation. Third, the levels of microRNA-9 (miR-9) and its precursor pri-miR-9-2 decreased in patient neurons but not in control neurons. The latter is likely because of reduced TDP-43, as shRNA-mediated TDP-43 knockdown in rodent primary neurons also decreased the pri-miR-9-2 level. The reduction in miR-9 expression was confirmed in human neurons derived from iPSC lines containing the more pathogenic *TARDBP* M337V mutation, suggesting miR-9 downregulation might be a common pathogenic event in FTD/ALS. These results show that iPSC models of FTD/ALS are useful for revealing stress-dependent cellular defects of human patient neurons containing rare TDP-43 mutations in their native genetic contexts.

## Introduction

Frontotemporal dementia (FTD), the second most common form of presenile dementia, and amyotrophic lateral sclerosis (ALS), a neurodegenerative disease that primarily affects motor neurons in the spinal cord and brain, are regarded as closely related conditions [1]. Genetic mutations and pathological proteins associated with both diseases include valosin-containing protein [2], [3], multivesicular body protein 2B [4], [5], ubiquilin 2 [6], chromosome 9 open reading frame 72 [7], [8], transactive response DNA-binding protein 43 (TDP-43) and fused in sarcoma (FUS) [9]–[13].

TDP-43 is a disease-related protein that links FTD and ALS. TDP-43 has two RNA recognition motifs and a glycine-rich region and is implicated in multiple aspects of RNA metabolism [14], [15]. Among these, TDP-43 has been linked to the processing of microRNAs (miRNAs) [16]–[18]. MiRNAs are small noncoding RNAs that regulate gene expression post-transcriptionally by degrading their target mRNAs or repressing their translation [19], [20]. Although potential involvement of miRNAs in neurodegeneration has been increasingly appreciated, information about their regulation and function in diseased human neurons remains scarce [21].

Modeling neurodegenerative diseases with TDP-43 pathology in animal models has been challenging. Although loss of nuclear TDP-43 and intracellular TDP-43 aggregates are common pathological features in neurons of FTD and ALS patients, disease-causing mutations in the gene are rare. Thus, in most patients, other contributing genetic factors appear to function upstream of TDP-43 during disease initiation and progression, which is difficult to model in animals. Moreover, the proper expression level of TDP-43 is critically important for its function. Ectopic expression of wildtype TDP-43 alone can be toxic, and TDP-43 itself is under complex autoregulation [22]–[25]. Deletion of TDP-43 in mice is lethal [26]–[28], highlighting its importance, but its conditional knockout shows disease–relevant phenotypes [29]–[31].

The ability to generate human induced pluripotent stem cells (iPSCs) from somatic cells [32] allows disease phenotypes of human neurons to be analyzed in the context of the unique genetic background of individual patients. Indeed, iPSCs have been generated from patients with ALS and other neurodegenerative diseases [33]–[40]. In this study, we generated iPSC lines from an FTD/ALS patient with *TARDBP* A90V mutation and from a healthy family member with no known mutations. We characterized neurons from this mutation and identified cell-specific phenotypes. Among these, we identified reduced miR-9 expression as a molecular defect that is downstream of TDP-43 and common to human neurons with *TARDBP* A90V or M337V mutations.

## Results

### Generation of patient-specific iPSC lines

Multiple iPSC lines derived from two patients were examined in this study. Two lines were previously generated from a 56-year old male with the *TARDBP* M337V mutation [35]. In this study, we also generated iPSC lines from a subject with FTD/ALS who was a patient at the Memory and Aging Center, University of California, San Francisco. The patient had an A90V mutation in *TARDBP*, and his skin biopsy was obtained at the age 75 years. To minimize the difference in genetic background, a skin biopsy was also obtained from a 65-year-old family member who had no TDP-43 mutations or clinical symptoms of FTD and lacked the A90V mutation. The patient and control were tested negative for the common FTD genes including *GRN*, *MAPT* and *C9ORF72*. The patient's clinical symptoms and family history are described in detail in File S1.

**Figure 1:**
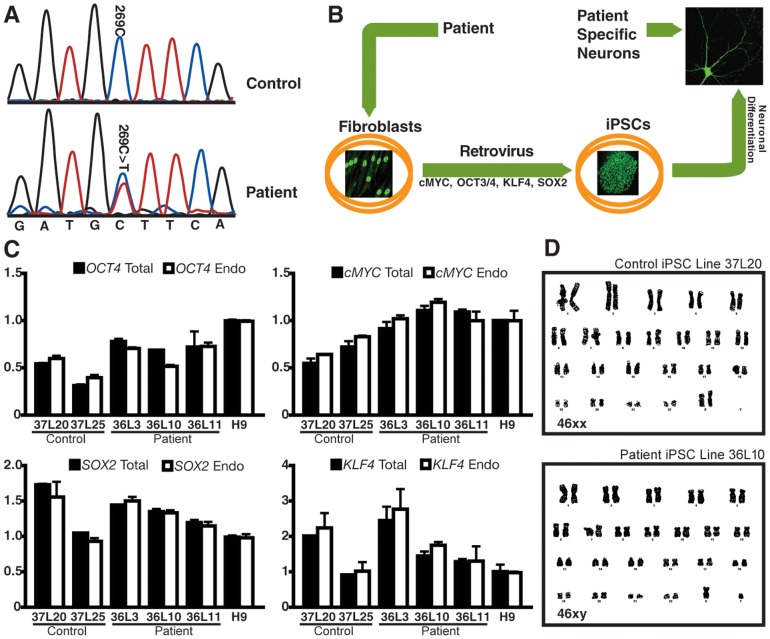
Generation and characterization of FTD/ALS patient-specific iPSC lines with the TDP-43 A90V mutation. (**A**) Sequencing of genomic DNA from patient fibroblasts confirmed the presence of the missense TDP43 A90V (c.269 C>T) mutation. (**B**) Schematic representation of the steps from the collection of patient fibroblasts to the derivation of postmitotic neurons. Fibroblasts, iPSCs and neurons were immunostained with TDP-43, NANOG and TUJ1 antibodies, respectively. (**C**) Quantitative RT-PCR analysis of the levels of total and endogenous expression of *OCT4*, *CMYC*, *SOX2*, and *KLF4* in iPSC and H9 ESC lines. Values were normalized to *GAPDH* levels. (**D**) No chromosomal abnormalities were found in any of the selected iPSC lines. The karyotypes of control line 37L20 and patient line 36L10 are shown.

Fibroblasts were derived from skin biopsies of both subjects. Genomic DNA was isolated from cultured fibroblasts, and the region flanking the expected *TARDBP* A90V mutation was PCR-amplified and sequenced to confirm the presence of the c.269C>T (A90V) mutation (Fig. 1A). The fibroblasts were reprogrammed with retrovirus expressing OCT4, SOX2, KLF4, and cMYC as described (Fig. 1B) [41]. After 4 to 5 weeks, iPSC colonies were manually picked and transferred to a Matrigel-coated plate. At least 20 clones from each individual were obtained for further characterization.

**Figure 2:**
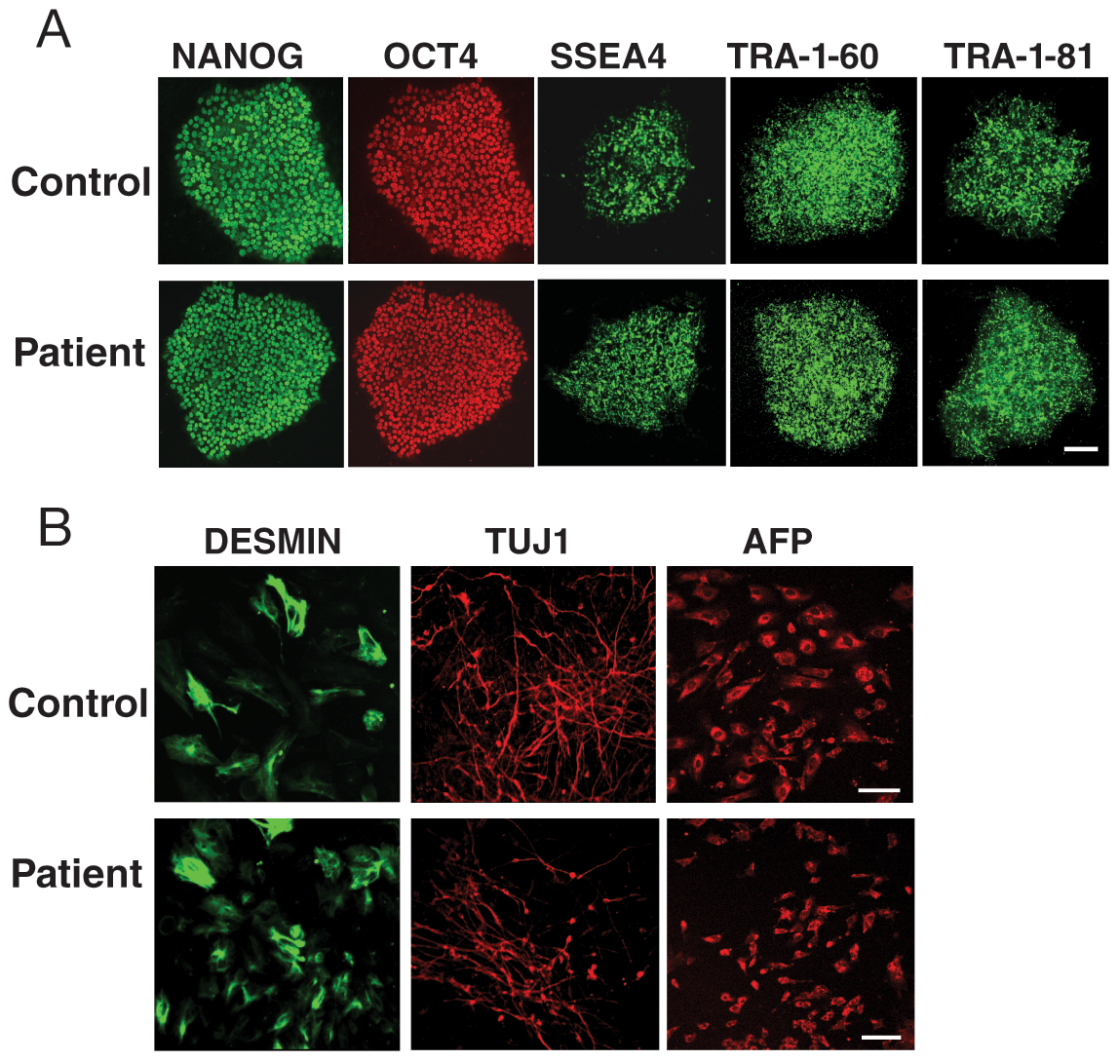
Verification of the pluripotency of the iPSC lines with the TDP-43 A90V mutation. (**A**) Fluorescence microscopy images of the expression of the pluripotency markers NANOG, OCT4, SSEA4, TRA-1-60, and TRA-1-81 in control (37L25) and patient (36L10) iPSC lines. Scale bar: 20 µm. (**B**) All iPSC lines differentiated into cells of the three germ layers, as shown by expression of desmin (mesoderm), TUJ1 (ectoderm), and alpha-fetoprotein (AFP, endoderm). These analyses indicate iPSC lines generated here are indeed pluripotent. Scale bar: 20 µm.

To identify fully reprogrammed iPSC lines, we performed several assays. First, to confirm that ectopically expressed reprogramming factors were effectively silenced; we used quantitative RT-PCR (qRT-PCR) to measure the endogenous and total levels (expressed from both the endogenous locus and the retroviral vector) of each reprogramming factor. The total level was comparable with that expressed from the endogenous locus, consistent with silencing of the transgene (Fig. 1C). Next, we selected 2 iPSC lines from the family member and 3 lines from the patient. All these lines maintained a normal karyotype (Fig. 1D and Fig. S1) and all continuously expressed pluripotent stem cell markers including NANOG, SSEA4, OCT4, TRA-1-60, and TRA-1-81 (Fig. 2A). We then examined the differentiation capability of the iPSCs *in vitro* through the formation of EBs. Spontaneous differentiation into derivatives of the three embryonic germ layers was confirmed after 1 week of cell culture (Fig. 2B). Altogether, these results indicate that the reprogramming was successful. The five selected iPSC lines were used for further studies below.

### Differentiation of iPSCs with TDP-43 mutations into functional neurons

**Figure 3:**
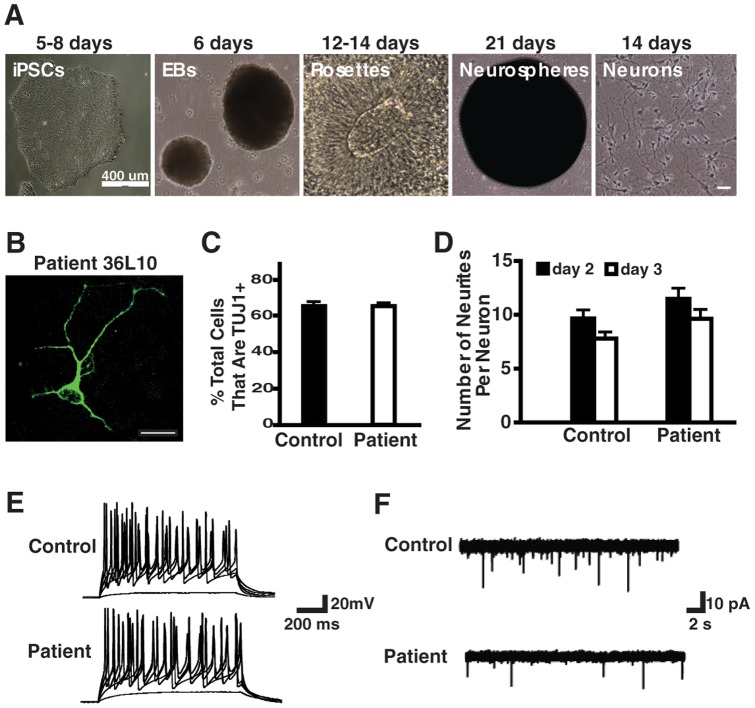
Characterization of iPSC-derived neurons with the TDP-43 A90V mutation. (**A**) Representative images of each of the five stages of the neural differentiation. The numbers of days cells are in each stage are listed on the top of each image. (**B**) Representative image of TUJ1 staining in patient neurons (36L10). (**C**) Percentage of TUJ1^+^ cells in control and patient neuronal cultures differentiated from iPSC lines 37L20, 37L25, 36L3 and 36L11. (**D**) Number of neurites in control and patient neurons differentiated from iPSC lines 37L20, 37L25, 36L3 and 36L10. (**E**) Sample recording of normal induced action potentials in response to somatic current injections in control and patient neurons differentiated from iPSC lines 37L20, 37L25, 36L10 and 36L11. (**F**) Spontaneous synaptic activity of control and patient neurons differentiated from iPSC lines 37L20, 37L25, 36L10 and 36L11.

The most prominent clinical phenotypes of the patient during 20 years of slow disease progression after the first diagnosis were all typical symptoms of FTD. To establish a human neuronal model of TDP-43-related FTD, we differentiated iPSCs with the *TARDBP* A90V and M337V mutations into postmitotic human neurons using a protocol for differentiating human embryonic stem cells into neural progenitor cells expressing the telencephalon marker BF1 [42], [43]. Briefly, EBs were formed and cultured in suspension before the induction of rosette formation with basic fibroblast growth factor 2 in serum-free medium containing N2 supplement for 12 days. Rosette cells were isolated and expanded in suspension culture for 21 days to form neurospheres. To differentiate dissociated neural progenitor cells into mostly postmitotic neurons, brain-derived neurotropic factor and glial cell line–derived neurotropic factor were included in the culture medium for 14 days (Fig. 3A).

After neuronal differentiation, about 65% of cells differentiated from control or patient iPSC lines with the *TARDBP* A90V mutation were immunopositive for the neuronal marker TUJ1 (Fig. 3B, C). A similar percentage of TUJ1-positive cells was obtained from neuronal differentiation of M337V and its control iPSC lines (data not shown). Moreover, the percentages of excitatory and inhibitory neurons were indistinguishable (Fig. S2), indicating the reproducibility of each differentiation process and the A90V mutation did not affect neuronal differentiation. The morphology and number of neurites were similar in neurons from the FTD/ALS patient and the family member (Fig. 3D). At 4 to 5 weeks after induction, all recorded neurons differentiated from control and patient iPSCs generated normal action potentials (Fig. 3E). We also determined whether control and patient iPSC neurons could form functional synapses. After terminal differentiation for 4 to 5 weeks, 80% of recorded control neurons, but only about 50% of patient neurons, showed spontaneous synaptic currents (Fig. 3F). Although the amplitude of spontaneous synaptic currents was similar (control neurons: 27.10±1.95 pA; patient neurons: 31.58±2.48 pA), the frequency of postsynaptic currents was lower in the patient neurons (0.09±0.01 versus 0.26±0.05 events/second) (Fig. 3F). These results suggest there might be a significant functional difference between control and patient neurons, which should be further investigated in the future.

### Patient Neurons with the *TARDBP* A90V Mutation Show Stress-Dependent Cellular and Molecular Phenotypes

**Figure 4:**
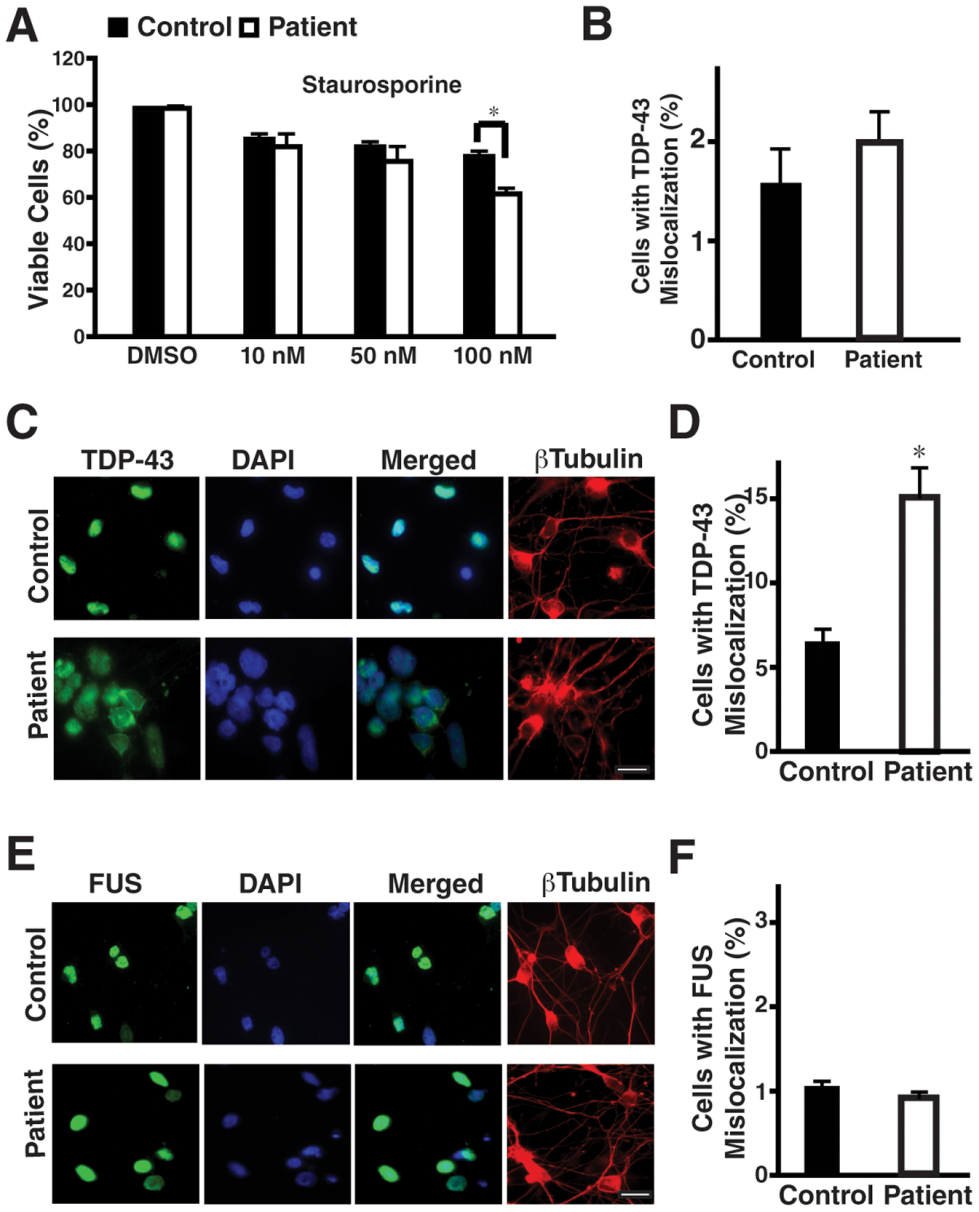
Patient neurons with the TDP-43 mutation are more susceptible to staurosporine and display increased TDP-43 mislocalization under stress. **(A)** Viability of control and patient neurons after exposure to10–100 nM staurosporine (STS) for 24 hours. More patient neurons die after treatment with 100 nM STS. Values are mean ± SEM. *: *p*<0.05 (Student's t-test). **(B)** The percentages of TUJ1^+^ control and patient neurons with mislocalized TDP43 without any stressor treatment. In Panels A and B, iPSC lines 37L20, 37L25, 36L10 and 36L11 were used. **(C, D)** Representative images of TDP-43 and TUJ1 staining in control and patient neurons differentiated from iPSC lines 37L20 and 36L10 after treatment with 100 nM staurosporine for 24 hours (C) and the percentage of TUJ1^+^ control and patient neurons showing mislocalized TDP-43 (D). After STS treatment, more patient neurons show TDP-43 cytoplasmic mislocalization. Some cells are non-neuronal cells, for instance, some cells with large nuclei are not labeled by MAP2. Values are mean ± SEM. *: *p*<0.05 (Student's t-test). **(E, F)** Representative images of FUS and TUJ1 staining in control and patient neurons differentiated from 37L20 and 36L11 after treatment with 100 nM staurosporine for 24 hours (E) and the percentage of TUJ1^+^ control and patient neurons differentiated from 37L20, 37L25, 36L10 and 36L11 showing mislocalized FUS (F). FUS localization is not affected by the *TARDBP* A90V mutation.

One of the challenges in using iPSCs to model age-dependent neurodegenerative disease is whether one can observe any disease-relevant phenotypes in cell culture. We hypothesized that under certain cellular stress, patient neurons would exhibit cellular and molecular phenotypes distinct from those of control neurons. First we examined the viability of neurons treated with different inducers of cellular stress. Patient and controls neurons were equally sensitive to sorbitol, an inducer of osmotic and oxidative stress (Fig. S3A) [44], sodium arsenite (Fig. S3B) and tBOOH (Fig S3C), both inducers of oxidative stress [45], [46]. However, more patient neurons died after treatment with 100 nM staurosporine (STS) (Fig. 4A), an inducer of apoptotic cell death [47].

Redistribution of TDP-43 from the nucleus to the cytoplasm is a characteristic of neurons in brain and spinal cord sections from FTD and ALS patients [9]. Immunostaining to detect the subcellular distribution of TDP-43 in patient neurons revealed that, under normal conditions, TDP-43 was present in the nucleus in the majority of cells (Fig. 4B). However, in response to100 nM STS, TDP-43 was mislocalized from nucleus to cytoplasm in more patient neurons than control neurons (Fig. 4C, 4D). FUS, another RNA binding protein associated with FTD and ALS [46], remained in the nucleus in both control and patient neurons after STS treatment (Fig. 4E, 4F).

**Figure 5:**
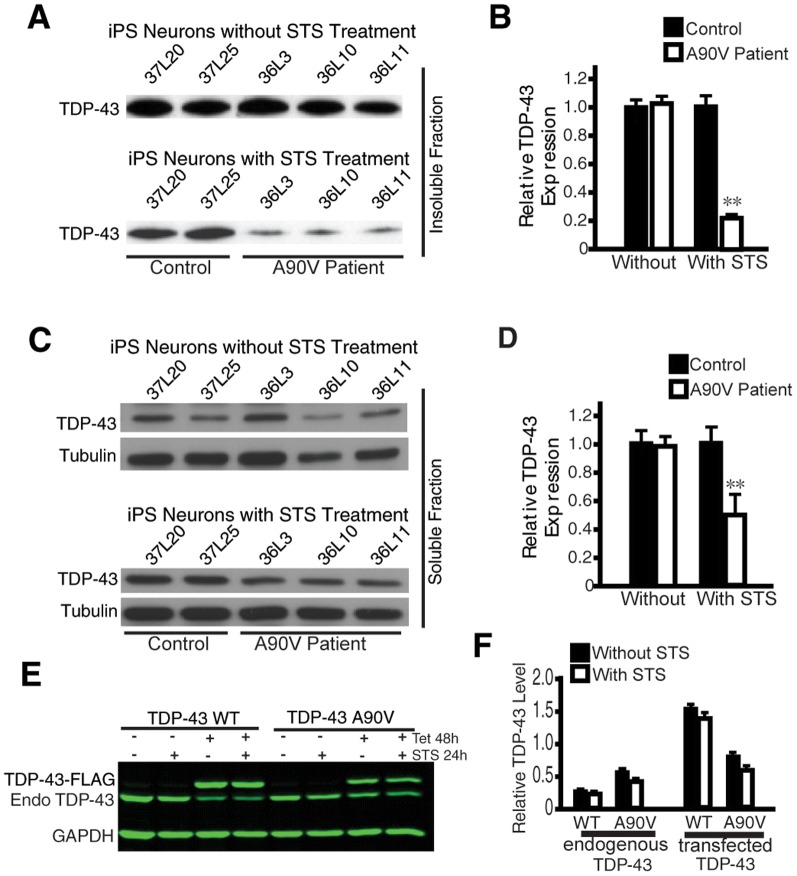
Cellular stress decreases TDP-43 levels in patient neurons with the TDP-43 A90V mutation. (**A**) The level of full length TDP-43 in the insoluble fraction of patient neurons remains the same as control neurons without STS treatment but decreases with STS treatment. (**B**) Quantification of the experiment in Panel A. (**C**) TDP-43 level in the soluble fraction of patient neurons decreases after STS treatment. (**D**) Quantification of the experiment in Panel C. The relative level of TDP-43 in each lane is normalized with the tubulin band and quantification is based on three independent experiments. (**E**) Western blot analysis of the levels of endogenous (Endo) and transfected TDP-43 in cell lines stably expressing tetracycline (tet)-inducible either wildtype (WT) TDP-43-Flag or TDP-43 A90V-Flag with or without STS treatment. The expression of transfected TDP-43 WT or TDP-43 A90V is induced by tetracycline. The A90V mutation alone is insufficient to decrease TDP-43 level in this assay. (**F**) Quantification of the experiment in Panel E. There is no statistical difference between samples treated with and without STS.

Increasing evidence suggests that either increased or decreased TDP-43 expression is associated with neurodegeneration [14], [15], [24], [30]. To examine TDP-43 expression in patient neurons under cellular stress, we measured TDP-43 in the insoluble and soluble fractions of patient neurons with the *TARDBP* A90V mutation treated with STS. The cells were extracted first with RIPA buffer and then with urea buffer to recover insoluble TDP-43, and analyzed by western blot. The TDP-43 level in the insoluble fraction was similar in untreated control and patient neurons with the *TARDBP* A90V mutation, but levels were significantly lower in patient neurons after STS treatment (Fig. 5A, B). Similarly, in the soluble fraction, the TDP-43 level was comparable in untreated patient and control neurons (Fig. 5C). However, after STS treatment, TDP-43 (Fig. 5D) but not FUS (Fig. S4A) was significantly lower in patient neurons with the *TARDBP* A90V mutation, consistent with the notion that loss of TDP-43 activity is also associated with neurodegenerative processes. This decrease is likely in part due to transcriptional regulation since *TDP-43* mRNA level is reduced in patient neurons after STS treatment (Fig. S4B). This decrease may not be contributed by the difference in protein stability between TDP-43 WT and TDP-43 A90V since STS treatment does not seem to affect the steady state level of transfected TDP-43 A90V compared with TDP-43 WT (Fig. 5E, F). Indeed, there is no intrinsic difference in the protein stability of transfected TDP-43 WT and TDP-43 A90V (Fig. S5A, B). Moreover, both transfected TDP-43 WT and TDP-43 A90V could effectively suppress the expression of endogenous TDP-43, indicating a normal autoregulatory function (Fig. S5C, D).

**Figure 6:**
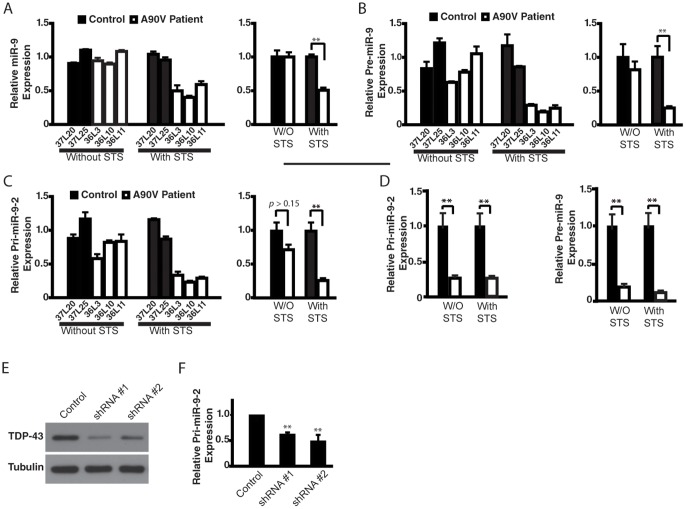
Expression of miR-9 and its precursors is reduced in patient neurons under stress. (**A**) Relative expression levels of mature miR-9 in neurons derived from two control and three patient iPSC lines containing the A90V mutation with or without STS treatment (left panel). The averages in control and patient neurons are shown on the right. (**B**) Expression of pre-miR-9. (**C**) Expression of pri-miR-9-2. The average of different lines from the same individual is set as 1. The results in A–C were confirmed from additional two independent neuronal differentiation experiments. (**D**) The relative expression levels of pri-miR-9-2 and pre-miR-9-2 in neurons derived from two patient iPSC lines containing the *TARDBP* M337V mutation was reduced. This result is based on two independent differentiation experiments. (**E**) TDP-43 levels were greatly decreased in primary mouse neurons transfected with two *TARDBP* shRNAs. (**F**) Expression of pri-miR-9-2 is decreased in primary mouse neurons transfected with *TARDBP* shRNA. Values are mean ± SEM. **: *p*<0.01 (Student's t-test).

TDP-43 has been implicated in the miRNA pathway [16], and alterations in the activity of several miRNAs have been associated with neurodegeneration [21]. Among them, miR-9, a brain-specific miRNA whose nucleotide sequence is highly conserved through evolution, is implicated in several neurodegenerative diseases and of great interest [49]. Indeed, the level of miR-9 (Fig. 6A) was significantly lower in patient neurons with the *TARDBP* A90V mutation than control neurons only after STS treatment. This decrease did not seem to be due to a defect in the miRNA-processing pathway, since STS also reduced the levels of both pre-miR-9-2 and pri-miR-9-2 in patient neurons (Fig. 6B, C). Moreover, pri-miR-124-1, the most abundant brain-specific miRNA, was not affected (Fig. S6A). Pri-miR-9-2 and pri-miR-124-1 were analyzed here because they are the most abundant among three miR-9 or miR-124 alleles, separately [50], [51]. In neurons with the *TARDBP* M337V mutation, the levels of pre-miR-9-2 and pri-miR-9-2 were also significantly lower after STS treatment than that in neurons derived from two control individuals (Fig. 6D). Interestingly, even without STS treatment, miR-9-2 expression was already lower in neurons with the *TARDBP* M337V mutation (Fig. 6D), consistent with the more pathogenic nature of the M337V mutation. To demonstrate that the decrease in miR-9a expression resulted directly from reduced TDP-43 function under cellular stress, we used mouse TDP-43-specific shRNAs (Fig 6E). TDP-43 knockdown in mouse primary cortical neurons indeed reduced the expression of pri-miR-9-2 (Fig. 6F) but not miR-124-1 (Fig. S6C).

## Discussion

To better understand the molecular pathogenic events in FTD, we used human neurons of telencephalon lineage differentiated from iPSCs with the *TARDBP* M337V mutation [35] or with the *TARDBP* A90V mutation as generated and characterized here. Using this system, we found that patient neurons with the *TARDBP* A90V mutation are less viable under cellular stress with the kinase inhibitor staurosporine treatment than control neurons. In these patient neurons, the percentage of cells with mislocalized cytoplasmic TDP-43 was increased. The expression of TDP-43 was also significantly decreased under cellular stress induced by STS. Moreover, miR-9 expression is significantly reduced in patient neurons with the *TARDBP* M337V mutation or in neurons with A90V mutation after STS treatment, revealing another stress-related molecular defect in patient neurons.

A major advantage of using iPSC-derived human neurons to study neurodegeneration is the presence of other patient-specific genetic factors in these cells. Several hundred genetic mutations may exist in each patient's genome and their collective contribution to disease process can be assessed. The A90V mutation is rare and considered to be a risk factor for FTD/ALS [52], [53]. Thus, examining the A90V mutation alone in animal models without other genetic modifiers may not be sufficient to reveal disease mechanisms. The cellular and molecular phenotypes observed here in iPSC-derived patient neurons are likely due to the overall genetic and epigenetic makeup of the patient including contributions from the A90V mutation. Although we used a family member as negative control to minimize the difference in genetic background, we understand the results obtained here from one patient need to be interpreted cautiously and we are aware of the challenges of generating more iPSC lines from other patients with the same rare mutation. So it remains to be seen whether neurons derived from other patients with the same or different TDP-43 mutations exhibit these cellular phenotypes under stress.

It is interesting to note that the A90V patient's disease progression after the initial diagnosis was unusually slow and lasted over two decades, while the partial loss of TDP-43 expression in the patient's iPSC-derived neurons occurred only after the treatment with a high concentration of STS. This decrease in TDP-43 level could be due to the effects of A90V mutation on nuclear import or in combination with other genetic factors. These findings support the hypothesis that the A90V mutation is pathogenic but with a slower kinetics. This observation stands in contrast to human neurons differentiated from patients with the M337V mutation where increased TDP-43 aggregates were observed even without stress treatment [35], [36], consistent with the notion that this mutation is more pathogenic.

Our results also indicate that miR-9 is specifically dysregulated in stressed patient neurons with the *TARDBP* A90V mutation, likely as a direct result of the reduced TDP-43 level observed in these neurons, as shRNA-mediated knockdown of TDP-43 in mouse primary cortical neurons also reduced miR-9 expression (Fig. 6). In human neurons containing the more pathogenic M337V mutation, miR-9 expression is also reduced even without STS treatment (Fig. 6). However, it is worth noting that human neurons in the normal culture conditions are already under cellular stress. Although total level of TDP-43 may increase in human neurons containing the *TARDBP* M337V mutation once the inter-patient variability is taken into account [36], a number of studies indicate that the M337V mutation results in the partial loss of normal TDP-43 function [18], [54], [55] and increased TDP-43 aggregates in human neurons [35], [36]. Thus, normal function of TDP-43 is compromised in human neurons containing the M337V mutation. Taken together, these findings support the notion that partial loss of TDP-43 normal function is also associated with neurodegeneration in FTD/ALS.

A role for TDP-43 in miRNA regulation has been demonstrated in previous studies [16]–[18], [56]. Similarly, partial loss of FUS function also results in a reduced miRNA biogenesis [57]. A prominent feature of TDP-43 pathology is the depletion of nuclear TDP-43 in many affected neurons, suggesting a possible significant pathogenic role for the loss of its normal nuclear function [9]. Moreover, mutations in another miRNA regulating protein FUS cause ALS [12], [13] and wild-type FUS forms inclusions in 5% of FTD cases [48]. Thus, misregulation of miRNAs and miR-9 in particular may be a common downstream molecular event of both disorders. TDP-43 regulates hundreds of targets [25], [58], [59]. To what extent miR-9 and specific miR-9 targets contribute to TDP-43 mediated neurodegeneration remains to be determined. The availability of different FTD/ALS patients-specific iPSC lines generated here and from other labs will undoubtedly further facilitate studies on the miRNA pathway and other pathogenic mechanisms.

## Materials And Methods

### Cell Culture and Generation of Human iPSCs

Skin biopsies were collected from a 75-year-old man who had a *TARDBP* missense mutation (A90V) and from an unaffected 65-year-old family member who lacked the mutation. The study was approved by the Institutional Review Board and Ethics Committees at the University of California, San Francisco (UCSF), and written informed consent was obtained in all cases. To protect patient family privacy, no additional information is presented here. Primary dermal fibroblasts derived from the explants were cultured in DMEM supplemented with 10% FBS, nonessential amino acids, and penicillin/streptomycin and reprogrammed into iPSCs using four transcription factors (*OCT3/4*, *SOX2*, *KLF4*, and *c-MYC)* as described (41). Five weeks after viral transduction, colonies were transferred to 12-well plates coated with Matrigel (BD Biosciences) and cultured in mTeSR1 medium (StemCell Technologies). For expansion, colonies were dissociated with Accutase and plated on 6-well plates. We also used iPSC lines from a 56-year-old man with the *TARDBP* M337V mutation and from two healthy controls [35].

### Karyotyping

Karyotyping analysis was carried out by the Cytogenetic Laboratory at the University of Massachusetts Memorial Medical Center (Worcester, MA). Cells were analyzed by standard G-banding technique, and results were interpreted by specialists in the Cytogenetic Laboratory.

### Neuronal Differentiation

iPSCs with either *TARDBP* A90V mutation generated in this study or the M337V mutation [35] were differentiated as described [37], [42] with some modifications. Embryoid bodies (EBs) were cultured in suspension for 6 days and attached to dishes coated with poly-L-ornithine/laminin. 12–14 days later, rosettes were collected and grown in suspension as neurospheres. After 3 weeks in culture, the neurospheres were dissociated with Accutase, and the neurons were plated on glass coverslips coated with poly-L-lysine/laminin. In most experiments, neurons 2-weeks after the neurosphere stage were used. For spontaneous differentiation in vitro, EBs were transferred to Matrigel-coated plates after 6-8 days as a floating culture for 8 days. Migrating cells from EBs were stained for markers of the three germ layers and analyzed by immunofluorescence microscopy. For the analysis on M337V neurons, two independent differentiation experiments were performed in which two M337V iPSC lines and one or three control iPSC lines derived from two control individuals were used.

### Immunocytochemistry

Cells were fixed in 4% paraformaldehyde for 10 minutes, permeabilized with 0.3% Triton X-100 for 10 minutes, and blocked with 10% BSA for 1 hour at room temperature. Cultures were then stained overnight at 4°C with goat anti-NANOG (1∶100, R&D, AF1997), mouse anti-OCT3/4 (1∶100, Santa Cruz Biotechnology, sc-5279), mouse anti-SSEA4 (1∶100, Abcam, ab16287), mouse anti-TRA-1-60 (1∶50, Chemicon, MAB4360), mouse anti-TRA-1-81 (1∶100, Chemicon, MAB4381), rabbit anti-desmin (Thermo Scientific; 1∶100, RB-9014-P0), mouse anti-α-fetoprotein (AFP, R&D Systems; 1∶200, MAB1368), rabbit anti-TDP-43 (1∶200, Proteintech Group, 10782-2-AP), mouse anti-TUJ1 (1∶500, Promega, G712A), rabbit anti-VGLUT1 (1∶500, Synaptic Systems,135303)and rabbit anti-GABA (1∶1000, Sigma, A2052). After incubation with the primary antibodies, the cells were incubated with fluorescence-conjugated secondary antibodies and imaged with a Nikon D-Eclipse C1 confocal microscope or an Olympus IX70 fluorescence microscope.

### MTT Assay

After 14 days in culture, human neurons were treated for 24 hours with the following stressors: staurosporine, t-butyl hydroperoxide, sodium arsenite, and DMSO. The sorbitol treatment was for 30 minutes. MTT assays were performed with CellTiter 96 Non-Radioactive Cell Proliferation Assay kit (Promega) according to the manufacturer's protocol. All experiments were conducted in triplicate.

### Western Blots

iPSC-derived neurons were collected and homogenized in lysis buffer (50 mM Tris-HCl, pH 7.5, 150 mM sodium chloride, 1% Nondidet P40, and 0.5% sodium deoxycholate, and protein inhibitor cocktail). Homogenates were centrifuged at 13,000 rpm at 4°C for 20 minutes, and protein concentration was determined with the Bradford Assay (Bio-Rad). Proteins (5 µg) were separated on a 10% polyacrylamide-SDS gel and transferred to a PVDF membrane. After blocking, the membrane was incubated with anti-hTDP-43 (1∶2,000, Proteintech Group, 10782-2-AP, or 1∶1,000, Bethyl A303-223A for Figure 5E and Figure S5) and anti-tubulin antibodies (1∶8,000; Sigma, T6199), and finally with anti-rabbit or anti-mouse secondary antibodies conjugated with horseradish peroxidase.

### Quantitative RT-PCR

Total RNA was extracted and purified with the miRNeasy kit (Qiagen) and the Turbo DNA-free kit (Ambion). RNA (300 ng) was used for reverse transcription reaction with TaqMan reverse transcription reagent kits (Applied Biosystems). mRNA and miRNA expression levels were quantified with SYBR Green Master Mix, TaqMan microRNA assays, or Taqman Universal PCR master mix (Applied Biosystems). Ct values for each sample and gene were normalized to *GAPDH* (mRNA) or U6 (miRNA). Primer sequences are shown in Table S1.

### Lentiviral Production and Mouse Primary Neuronal Cultures

HEK293T cells were transfected with a mix of 2.6 µg plasmids expressing TDP-43-specific shRNA (Sigma), 26 µl lentiviral packaging mix (Sigma), and 16 µl FuGENE 6 (Promega). Thirty-six to forty-eight hours after transfection, medium containing viruses was filtered and centrifuged at 23,000 rpm for 2 hours at 4°C.The supernatant was removed, and the pellet containing the lentiviral particles was dissolved in 100 µl HBSS overnight and stored at −80°C. Primary neuronal cultures were obtained from C57/B6 mice (E18). Cells were plated at a density of 70,000 cells/well in 12-well plates and transduced with lentiviral particles after 3 days in vitro.

### Electrophysiology

Whole-cell perforated patch recordings were performed on FTD/ALS (*n* = 16) and control (*n* = 15) 4- to 5-week-old neurons grown in neuron medium containing 0.5% FBS on acid-etched coverslips. The recording micropipettes (tip resistance 3 to 6 MΩ) were tip-filled with internal solution composed of 115 mM K-gluconate, 4 mM NaCl, 1.5 mM MgCl_2_, 20 mM HEPES, and 0.5 mM EGTA (pH 7.4) and back-filled with the same internal solution containing 200 µg/ml amphotericin B (Calbiochem). Recordings were made with an Axopatch 200B amplifier (Axon Instruments). Signals were sampled at 10 kHz and filtered at 2 kHz. Whole-cell capacitance was fully compensated; series resistance was uncompensated but monitored during the experiment by the amplitude of the capacitive current in response to a 5-mV pulse. The bath was constantly perfused with fresh HEPES-buffered saline composed of 115 mM NaCl, 2 mM KCl, 10 mM HEPES, 3 mM CaCl_2_, 10 mM glucose, and 1.5 mM MgCl_2_ (pH 7.4). For voltage-clamp recordings, cells were clamped at −60 to −80 mV; Na^+^ currents and K^+^ currents were stimulated by voltage step depolarization. Command voltage varied from −50 to 20 mV in 10-mV increments. For current-clamp recordings, induced action potentials were stimulated with current steps from −0.2 to 0.5 nA. All recordings were performed at room temperature.

### Stable Cell Lines with Inducible TDP-43 Expression

Wildtype TDP-43 cDNA (HindIII-BamHI) was generated by PCR amplification and 2XFLAG tag was added at its C-terminal using primers (5′-GCGAAGCTTATGTCTGAATATATTCGGGTAAC-3′ and 5′-GCGGATCCTTACTTGTCATCGTCGTCCTTGTAGTCCTTGTCATCGTCGTCCTTGTAGTC CATTCCCCAGCCAGAAGACTTAG-3′). The TDP-43 PCR fragment was inserted into the polylinker of pcDNA5-TetO (Invitrogen) to make TDP-FLAG plasmid. A90V mutation was made by primer based Quick Change Method (Stratagene). 2 µg TDP-FLAG or A90V-FLAG constructs were transfected into HEK293 cells together with 0.2 µg of pOG44 using 7 µl of TransIT-293 (Mirus). Cell lines stably expressing TDP-FLAG or A90V-FLAG were generated by treating the cells with several rounds of Blasticidin (15 mg/ml) and Hygromycin (100 mg/ml) selection. To induce transgene expression, tetracycline (Tet; 500 ng/ml) (Sigma) was added at different time points in the absence or presence of STS.

To examine TDP-43 stability, 500 ng/ml of tetracycline was added to the stable cell lines to induce the expression of TDP-FLAG or A90V-FLAG. After 48 h of induction, cells were washed twice by 1XPBS and then replenished with fresh media without tetracycline. Cells were collected at different time points (4–48 hrs) after removing tetracycline. Harvested cells were lysed with RIPA buffer or trizol (Ambion) to get whole cell protein extract or total RNAs separately.

### Statistical Analysis

Values are presented as mean ± SEM. Statistical significance was assessed by ANOVA or *t* test. *P*<0.05 was considered significant.

## Supporting Information

Figure S1 **Karyotyping results show no chromosomal abnormalities in control iPSC line 37L25 or patient iPSC lines 36L11 and 36L3.**
(TIF)

Figure S2 **Neuronal subtypes in iPSC-derived neuronal cultures.** Most neurons are excitatory neurons and about 10% are inhibitory neurons. The presence of the A90V mutation does not affect the excitatory/inhibitory neuron ratio.
(TIF)

Figure S3 **Cell viability does not differ in control and patient neurons treated with 0.4 M sorbitol for 30 minutes (A), sodium arsenite (SA) (B) or t-butyl hydroperoxide (tBOOH) (C). **Quantification of cell viability in control and patient neurons after treatment with different stressors.
(TIF)

Figure S4 **Cellular stress does not decrease FUS levels in patient neurons but decreases** ***TDP-43*** **mRNA.** (**A**) FUS level in the soluble fraction of control and patient neurons with STS treatment. Quantification of the experiment is shown on the right. (**B**) *TDP-43* mRNA is decreased in patient neurons after STS treatment as measured by qRT-PCR.
(TIF)

Figure S5 **The protein stability and auto-regulation of the wildtype and A90V mutant TDP-43.** (**A**) Protein level of transfected TDP-43 in TDP-43 or TDP-43 A90V-Flag stable cell lines at different time point after tetracycline treatment. Endo TDP-43: endogenous TDP-43. (**B**) Quantification of the experiment in panel A. Values was normalized to transfected TDP-43 at 48 h after tetracycline treatment. (**C**) Induction of transfected either TDP-43 WT or TDP-43 A90V at different time points leads to gradual inhibition of endogenous TDP-43 (Endo TDP-43) expression. There is no statistical difference in the extent of autoregulation. (**D**) Quantification of the experiment in panel D. In this experiment, the sample collected at 48 hr after tetracycline induction appears to have higher protein level of TDP-43 WT than TDP-43 A90V, which is due to higher transfection since the wildtype mRNA is also higher (not shown).
(TIF)

Figure S6 **Expression of miR-124 does not change in patient and control neurons treated with STS or in primary mouse neurons after TDP-43 knockdown.** (**A**) Expression of pri-miR-124-1 in neurons derived from two control and three patient iPSC lines with or without STS treatment. (**B**) Average expression of pri-miR-124-1 in control and patient neurons. (**C**) Expression of pri-miR-124-1 in primary mouse neurons transfected with *TDP-43* shRNA.
(TIF)

Table S1 Primer sequences used in this study.
(DOC)

File S1 **Patient information.** The patient's clinical symptoms and family history are described in detail.
(DOCX)
