# Supplementary figures and images for: Downregulation of MicroRNA-9 in iPSC-Derived Neurons of FTD/ALS Patients with TDP-43 Mutations
Source: PLoS One. 2013 Oct 15;8(10):e76055. doi: 10.1371/journal.pone.0076055 (PMC3797144; doi:10.1371/journal.pone.0076055)

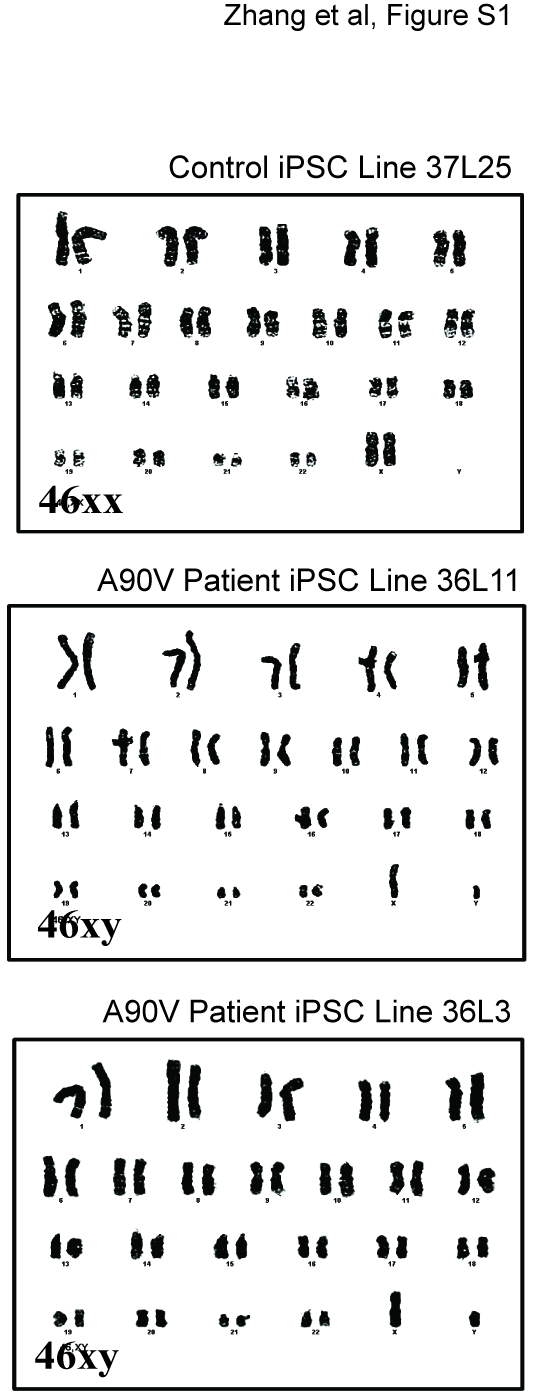

Supplement: Figure S1 — Karyotyping results show no chromosomal abnormalities in control iPSC line 37L25 or patient iPSC lines 36L11 and 36L3. (TIF) [file pone.0076055.s001.tif]

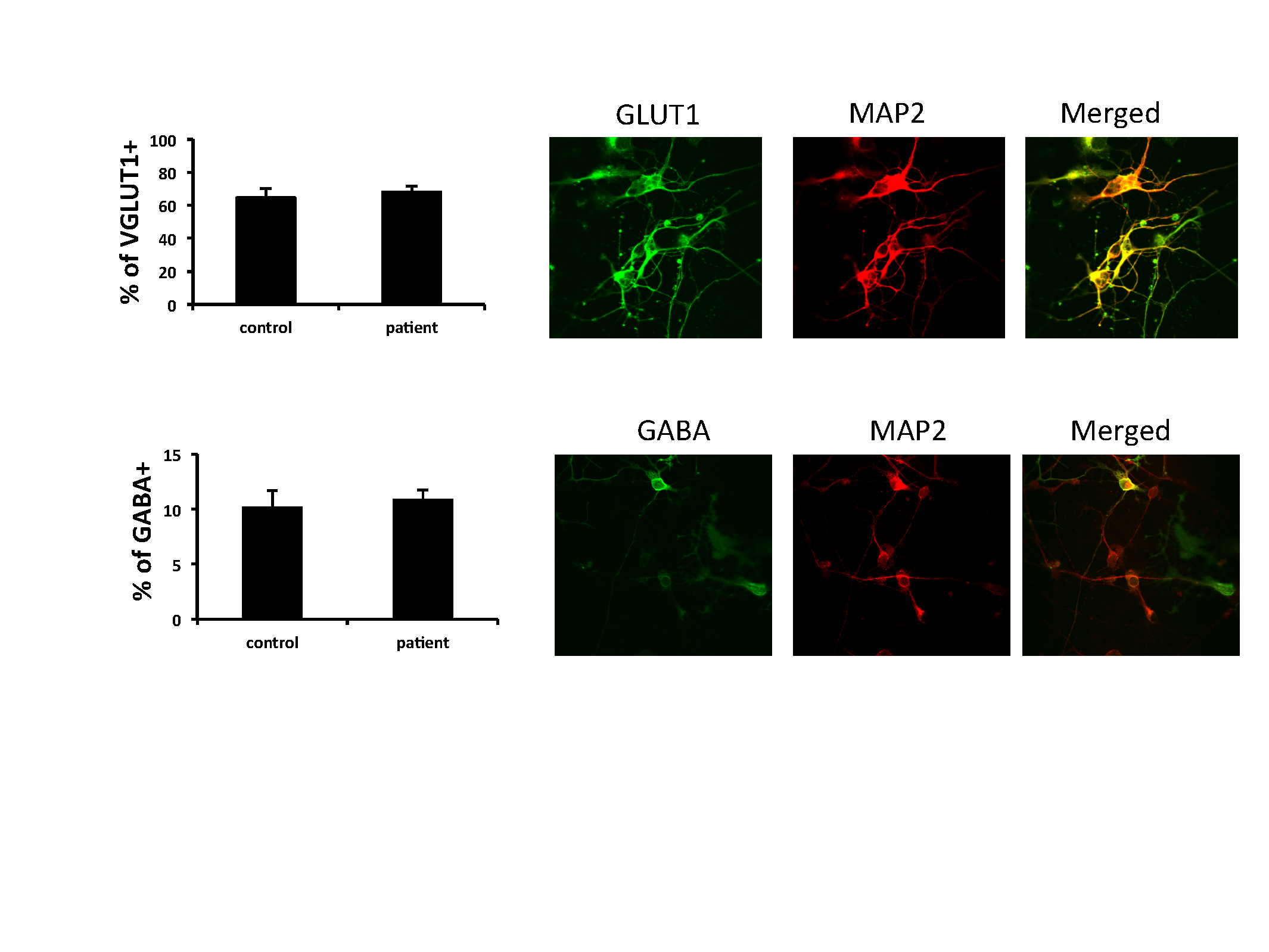

Supplement: Figure S2 — Neuronal subtypes in iPSC-derived neuronal cultures. Most neurons are excitatory neurons and about 10% are inhibitory neurons. The presence of the A90V mutation does not affect the excitatory/inhibitory neuron ratio. (TIF) [file pone.0076055.s002.tif]

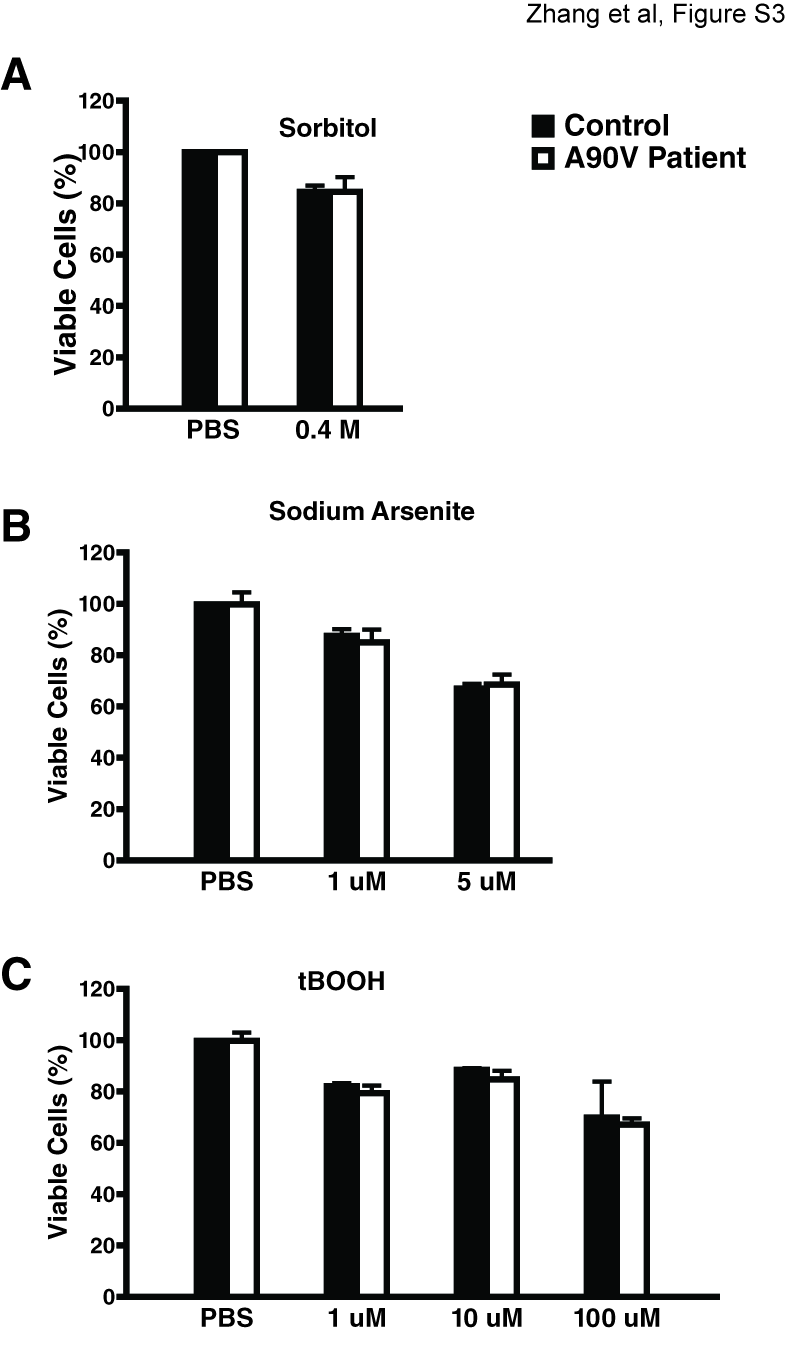

Supplement: Figure S3 — Cell viability does not differ in control and patient neurons treated with 0.4 M sorbitol for 30 minutes (A), sodium arsenite (SA) (B) or t-butyl hydroperoxide (tBOOH) (C). Quantification of cell viability in control and patient neurons after treatment with different stressors. (TIF) [file pone.0076055.s003.tif]

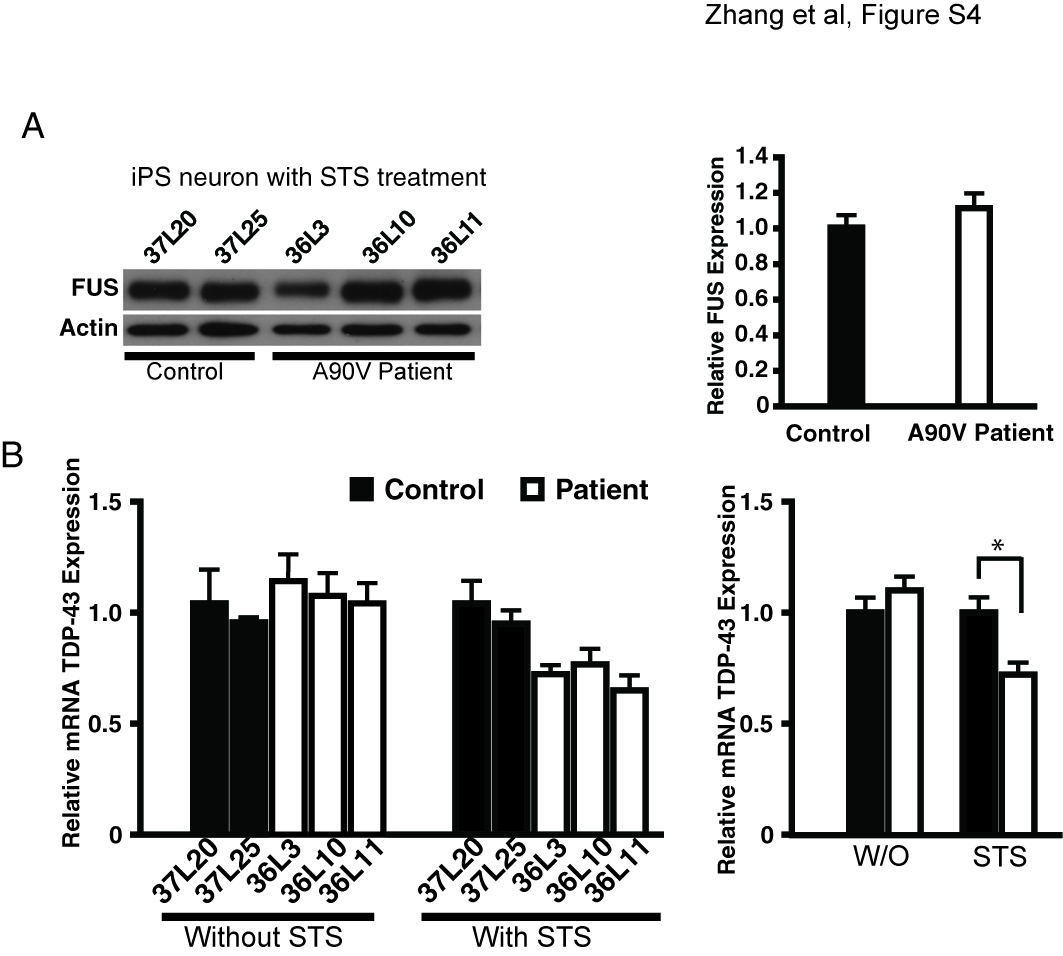

Supplement: Figure S4 — Cellular stress does not decrease FUS levels in patient neurons but decreases TDP-43 mRNA. (A) FUS level in the soluble fraction of control and patient neurons with STS treatment. Quantification of the experiment is shown on the right. (B) TDP-43 mRNA is decreased in patient neurons after STS treatment as measured by qRT-PCR. (TIF) [file pone.0076055.s004.tif]

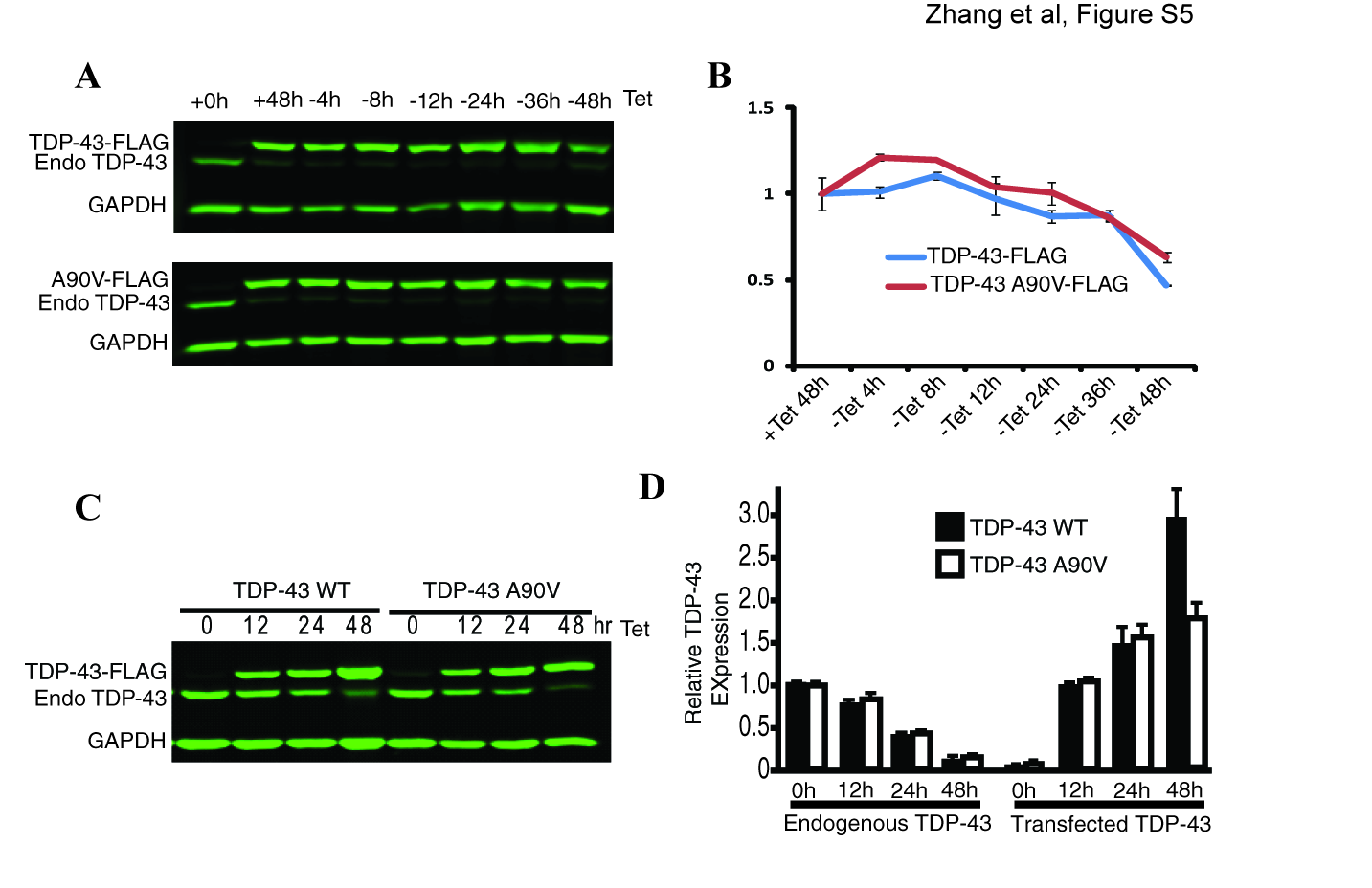

Supplement: Figure S5 — The protein stability and auto-regulation of the wildtype and A90V mutant TDP-43. (A) Protein level of transfected TDP-43 in TDP-43 or TDP-43 A90V-Flag stable cell lines at different time point after tetracycline treatment. Endo TDP-43: endogenous TDP-43. (B) Quantification of the experiment in panel A. Values was normalized to transfected TDP-43 at 48 h after tetracycline treatment. (C) Induction of transfected either TDP-43 WT or TDP-43 A90V at different time points leads to gradual inhibition of endogenous TDP-43 (Endo TDP-43) expression. There is no statistical difference in the extent of autoregulation. (D) Quantification of the experiment in panel D. In this experiment, the sample collected at 48 hr after tetracycline induction appears to have higher protein level of TDP-43 WT than TDP-43 A90V, which is due to higher transfection since the wildtype mRNA is also higher (not shown). (TIF) [file pone.0076055.s005.tif]

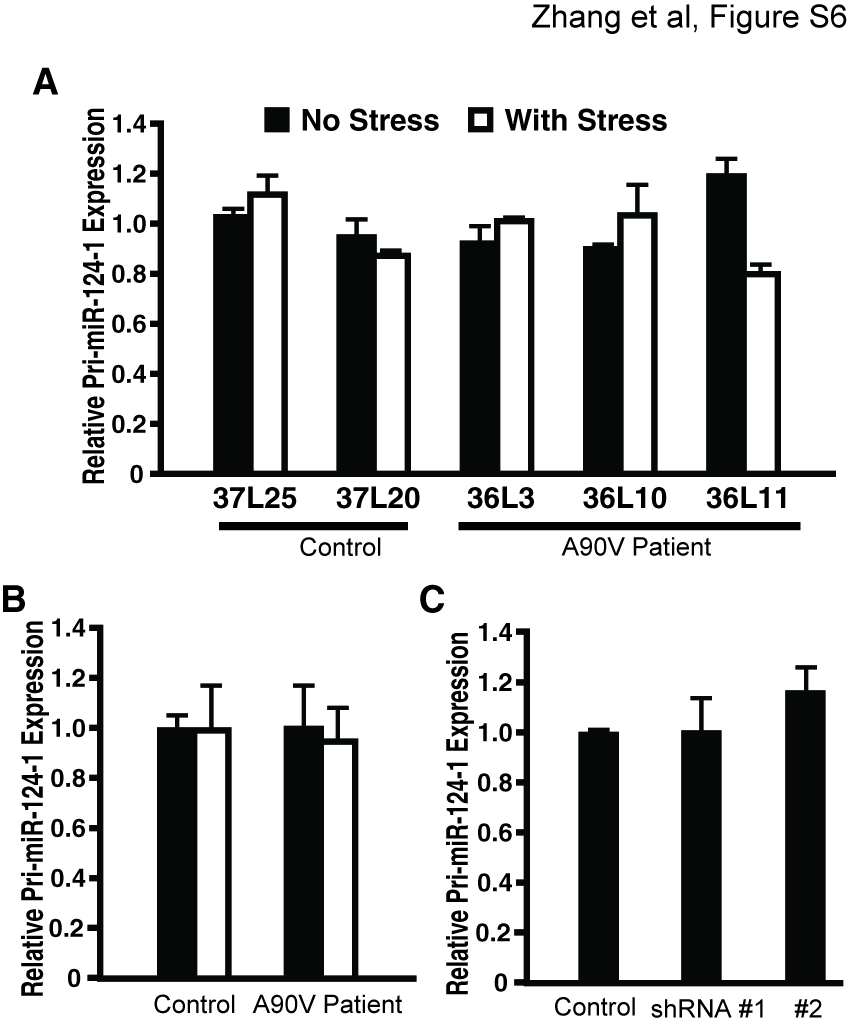

Supplement: Figure S6 — Expression of miR-124 does not change in patient and control neurons treated with STS or in primary mouse neurons after TDP-43 knockdown. (A) Expression of pri-miR-124-1 in neurons derived from two control and three patient iPSC lines with or without STS treatment. (B) Average expression of pri-miR-124-1 in control and patient neurons. (C) Expression of pri-miR-124-1 in primary mouse neurons transfected with TDP-43 shRNA. (TIF) [file pone.0076055.s006.tif]
