# Supplementary material for: Downregulation of MicroRNA-9 in iPSC-Derived Neurons of FTD/ALS Patients with TDP-43 Mutations
Source: PLoS One. 2013 Oct 15;8(10):e76055. doi: 10.1371/journal.pone.0076055 (PMC3797144; doi:10.1371/journal.pone.0076055)
Supplement: Table S1 — Primer sequences used in this study. (DOC) [file pone.0076055.s007.doc]

| Mouse Pri-miR9-2 | forward | GTGAGGGAAGCGAGTTGTTATC |
| --- | --- | --- |
|  | reverse | CCTCGGTGACCTTGAAGGAGTTTTTAC |
| Mouse pri-miR-124-1 | forward | cctccctttctttccaccttt |
|  | reverse | gagagggaggatgggagaa |
| Mouse U6 | forward | CACGAGCGAAGCCGTCGTG |
|  | reverse | AAAAATATGGAACGCTTCAC |
| Sox2 total | forward | AGCTACAGCATGATGCAGGA |
|  | reverse | GGTCATGGAGTTGTACTGCA |
| Oct4 endo | forward | gtactcctcggtccctttcc |
|  | reverse | caaaaaccctggcacaaact |
| Human GAPDH | forward | TGCACCACCACCTGCTTAGC |
|  | reverse | GGCATGGACTGTGGTCATGAG |
| Human Pri-miR9-2 | forward | GCTGTATGAGTGTATTGGTCTTCA |
|  | reverse | CCTGACCTTTCTGGTTTTTACTGT |
| Human Pre-miR9-2 | forward | GAAGCGAGTTGTTATCTTTGGTT |
|  | reverse | TGAAGGAGTTTTTACTTTCGGTTA |
