# Supplementary material for: Downregulation of MicroRNA-9 in iPSC-Derived Neurons of FTD/ALS Patients with TDP-43 Mutations
Source: PLoS One. 2013 Oct 15;8(10):e76055. doi: 10.1371/journal.pone.0076055 (PMC3797144; doi:10.1371/journal.pone.0076055)
Supplement: File S1 — Patient information. The patient's clinical symptoms and family history are described in detail. (DOCX) [file pone.0076055.s008.docx]

**Patient Information**

His illness began at age 56 years with personality changes and his disease progression was exceptionally slow. Over the following two years, his work performance had declined. He engaged in compulsive behaviors, including rummaging through dumpsters to collect discarded recyclable items as well as compulsive walking, coin sorting, and card playing. He hoarded food and overate sweet foods. Eight years after the first symptoms, he became socially disinhibited with a propensity to approach strangers with intrusive, inappropriate comments on their age, weight, or sexual characteristics. Eleven years after the first symptoms, he developed shortness of breath, fasciculations of the arms, and weakness of proximal muscles. Twenty years after the first symptoms and 2 years before his death, he was diagnosed with ALS.

The family history was notable for a diagnosis of obsessive-compulsive disorder (OCD) in his father in his 50s, followed by a diagnosis of dementia in his 60s. Four paternal aunts were diagnosed with dementia in their 60s, and the paternal grandfather had an unspecified dementia.
